# Intravascular ultrasound assessment of the effect of laser energy on the arterial wall during the treatment of femoro-popliteal lesions: a CliRpath excimer laser system to enlarge lumen openings (CELLO) registry study

**DOI:** 10.1007/s10554-017-1248-2

**Published:** 2017-09-26

**Authors:** Kayode O. Kuku, Hector M. Garcia-Garcia, Edward Koifman, Alexandre H. Kajita, Sameer Desale, Viana Azizi, Gebremedhin Melaku, Anh Bui, Yael F. Meirovich, Solomon Beyene, Aaphtaab Dheendsa, Blaine Schneider, Ron Waksman, On behalf of the CELLO study investigators

**Affiliations:** 1 MedStar Cardiovascular Research Network, MedStar Washington Hospital Center, Washington, DC USA; 2 MedStar Georgetown University Hospital, Washington, DC USA; 3 MedStar Health Research Institute, Hyattsville, MD USA; 4 Spectranetics Corporation, Maple Grove, Minnesota USA; 5 Division of Interventional Cardiology, MedStar Washington Hospital Center, 110 Irving Street NW, Washington, DC 20010 USA

**Keywords:** Excimer laser atherectomy, Intravascular ultrasound, Peripheral arterial disease, Femoro-popliteal, Dissections

## Abstract

The CliRpath Excimer Laser System to Enlarge Lumen Openings (CELLO) registry included patients treated with modified excimer laser catheters for the endovascular treatment of peripheral artery disease affecting the superficial femoral artery (SFA) and proximal popliteal artery. The aim of this study was to assess, via intravascular ultrasound (IVUS) the dissections in the vessel wall following treatment with the laser catheters. IVUS grayscale images from the CELLO registry were systematically reviewed for dissections in the treated vessel segments by two investigators. Images from 33 patients; 66 pullbacks (1867 IVUS frames in 2 phases), were successfully matched frame-to-frame to evaluate identical segments of the treated vessels in the two phases; post-2 mm Turbo-Elite laser pilot channel creation and post Turbo-Booster laser atherectomy. Dissections were categorized as; (1) intimal, (2) medial, (3) intramural hematoma, and (4) adventitial according to the ACC Clinical Expert Consensus Document classification of dissections. An average of 57 frames was evaluated per pullback, giving a total of 3734 frames (1867 matched for pre-ablation (post channel creation) and post-ablation phases). Treatments with the modified Excimer laser catheters resulted in a significant increase in lumen area of 5.5 ± 3.2-mm^2^ (95% CI 4.3–6.8, p < 0.0001) and reduction in plaque plus media volume of −10.6 ± 36.0 mm^3^ (95% CI −25.8 to 4.6, p = 0.1619) whilst giving rise to mainly intramural hematoma formations post Turbo-Booster laser treatment in 55% of frames assessed and 24% medial dissections with less than 1% adventitial disruption. The Excimer laser based Turbo-Booster treatment of peripheral artery lesions resulted in significant plaque debulking and increased lumen diameter with negligible degree of adventitial layer injury.

## Introduction

The excimer laser was developed about 40 years ago [1] for use in debulking atherosclerotic lesions in coronary arteries. Over the last decade, devices utilizing this technology have been refined and have demonstrated benefit in the treatment of peripheral arterial disease (PAD) due to the capacity of the laser for controlled atheroablation with short-absorption depths [2]. These results led to approval by the Food and Drug Administration (FDA) of Excimer laser-guided photo-ablation as an endovascular debulking modality in the treatment of PAD.

Femoropopliteal (FP) atherosclerotic lesions affect a mechanically dynamic vascular segment and are responsible for the majority of cases of lower-extremity PAD. Laser atherectomy has been shown to be beneficial in the treatment of FP diseases, but despite its increasing viability, restenosis rates associated with vascular procedures in general remain a concern due to vessel wall trauma that may occur during these treatments [3, 4]. The CliRpath Excimer Laser System to Enlarge Lumen Openings (CELLO) multicenter registry was designed to evaluate the safety and efficacy of modified Excimer Laser catheters for the endovascular treatment of peripheral artery disease affecting the superficial femoral and proximal popliteal arteries [5].

The aim of this post-hoc analysis was to quantitatively assess the intravascular ultrasound images from the CELLO registry patients for arterial wall dissections following treatment with the modified excimer laser catheters.

## Methodology

### Study design

The CELLO registry study design, eligibility criteria, end points, and details of the investigational device have been previously described in detail [5]. In brief, the CELLO registry was a multicenter prospective non-randomized trial which included 65 patients with mild to severe intermittent claudication secondary to peripheral artery disease (PAD) from 17 investigational sites in the United States. The study served as the basis for the clearance of the Turbo-Booster laser system by the United States Food and Drug Administration (FDA) in 2007. Patient demographics, clinical history as well as the baseline characteristics of treated lesions were collected from the registry.

The 8-F Turbo-Booster laser guide catheter (Spectranetics Corporation, Colorado Springs, CO, USA) consists of a modified guide catheter and a 2.0 mm Turbo-Elite laser catheter. A pilot channel was created through the lesion with the Turbo-Elite laser catheter followed by further debulking of the lesion with the Turbo-Booster system. Laser actuation occurred only during the ante-grade movement of the catheter through the artery and IVUS images were collected after both the pilot channel creation and Turbo-Booster treatment using a 40 MHz mechanical rotating IVUS Catheter [5].

### Intravascular ultrasound assessment

All IVUS assessment was carried out using computerized software Qivus 3.0 (Medis medical imaging systems) and Echoplaque (INDEC Medical Systems, Santa Clara, CA, USA) at the MedStar Cardiovascular Research Network, Invasive Coronary Imaging Core Laboratory. All available matching grayscale IVUS images were quantitatively reviewed by two investigators (H.G. and K.K.) to assess for the degree of change in the normal vessel anatomy in the two phases. Further reviews of the images were carried out in a conference setting in the presence of a third investigator (A. K) to arrive at consensus. The image frames were systematically evaluated at consecutive intervals of 0.5 mm within a 20–30 mm segment surrounding the site of the minimal lumen cross-sectional area -MLA- previously quantitatively assessed. The length was pre-specified and extended proximal and distal to the MLA, based on the calculated differences observed in lumen area in adjoining segments and wit consideration that the laser actuation occurred only during ante-grade movement of the catheter through the arteries.

The quantitative IVUS analyses of vascular, luminal and plaque dimensions for this cohort of patients previously reported as part of the original CELLO registry report were performed within a 10-mm volumetric area surrounding the site of minimal lumen cross-sectional area (MLA).

The impact of the laser treatment on the vessel wall, post-Turbo-Elite and post-Turbo-Booster, was categorized according to vessel wall dissections/disruptions classification as outlined in the American College of Cardiology Clinical Consensus Document on Standards for Acquisition, Measurement and Reporting of Intravascular Ultrasound Studies [6]. Disruptions to the vessel were evaluated qualitatively and quantitatively throughout the selected vessel segment by accurately pairing both phases of the IVUS pullbacks with the aid of vascular and perivascular markings (e.g., side branches, venous structures, calcific and fibrotic deposits and known pullback speeds. Dissections were classified as follows: (a) intimal dissection, (b) medial dissection, (c) intramural hematoma, and, (d) adventitial/extra-medial injury. Intimal dissection was defined as a dissection with a tear limited to the intima or atheroma, and not extending to the media. Medial dissection was defined as a dissection that extended into the media (and the angle of disruption measured). Intramural hematoma was a medial dissection distinguished by the appearance of blood accumulation within the medial space (entry or exit points may or may not be obvious). Extra-medial or adventitial injury was defined as a dissection extending beyond the media with blood visible in the perivascular tissue [6]. A total of 66 pullbacks (33 pairs) were successfully matched for assessment.

Frame-by-frame qualitative analysis was carried out prior to scoring of the images to account for clustering of the frames.

### Statistical analysis

The baselines clinical characteristics of the 33 patients were analyzed on a patient level while the IVUS information was analyzed on both patient and image frame level. The statistical analysis was performed using SAS software, Version 9.4 (SAS Institute, Cary, NC, USA). Continuous variables were presented as mean and standard deviation or median based on the distribution and categorical variables were presented as frequencies and percentages. No adjustments for patient of frame clustering data were performed in the statistical analysis. The paired test of differences was employed in comparison analysis and results were presented as least square means with 95% confidence intervals and a p value of < 0.05 was considered as significant.

## Results

**Fig. 1:**
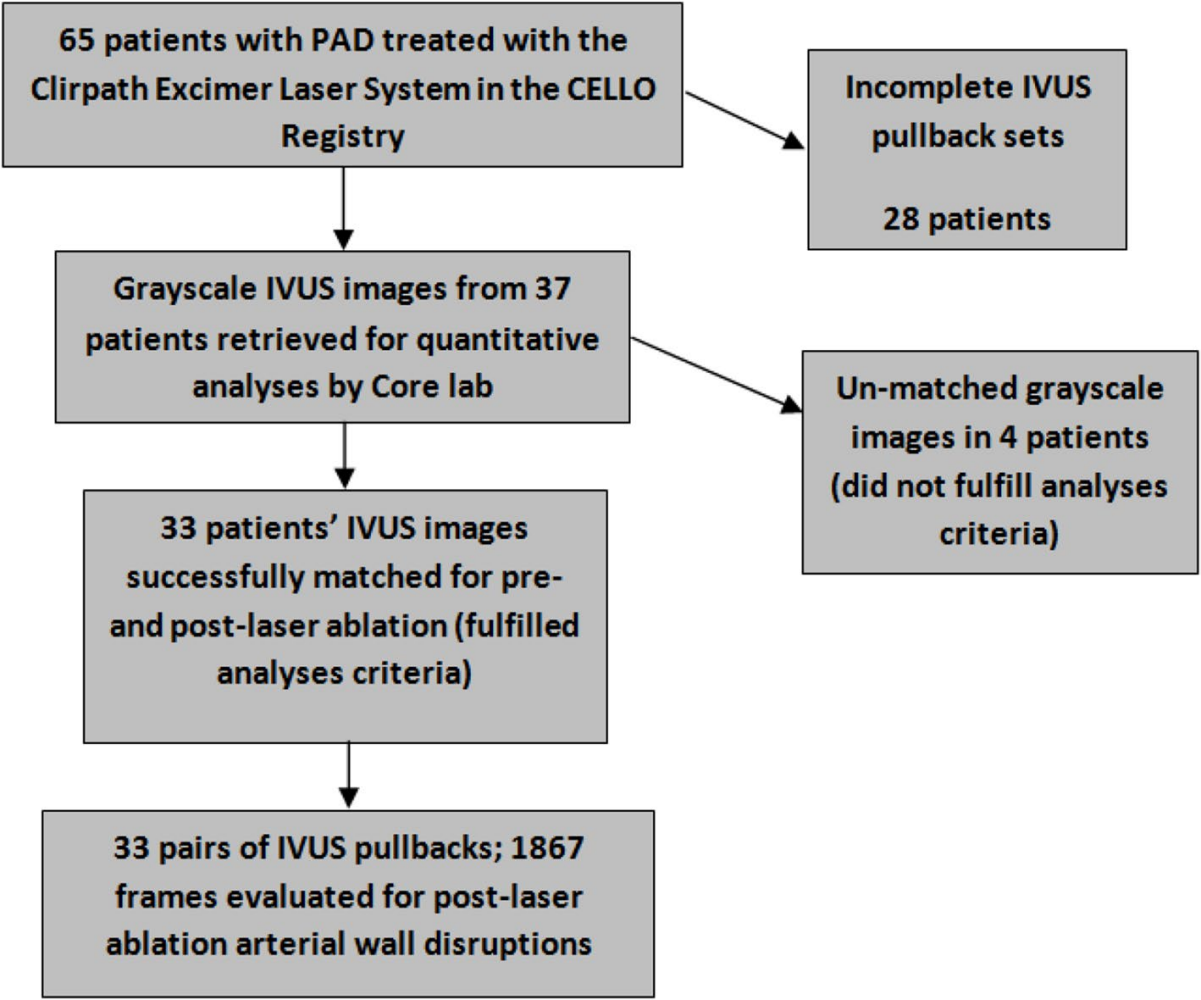
Study diagram-a total of 33 patients’ pullbacks (post-2 mm channel creation and post-Turbo-Booster pairs) fulfilled the criteria for objective post-hoc assessment for this sub-study

Among the 65 patients in the pivot study, 74 matching pullbacks from 37 patients treated for PAD affecting the SFA and proximal popliteal artery with Turbo-Elite and Turbo-Booster were successfully retrieved. Thirty-three patients met the criteria (availability of matching pair of post channel creation/pre- and post-laser ablation grayscale IVUS) for the analyses (Fig. 1).

### Clinical and procedure characteristics

The baseline clinical and procedural characteristics of the 33 patients and lesions are summarized in Table 1. The patients were predominantly elderly men (70% males) and the medical history and PAD risk factors were largely typical for this group of patients. The most common co-morbidities were hyperlipdemia (90.9%), hypertension (87.9%), smoking history (84.8%), and coronary artery disease (63.6%).

**Table 1:** Baseline clinical and procedural characteristics

Demographics (n = 33)	
Age, years	68.1 ± 9.7
Men, n (%)	23 (69.7)
Medical history	
Diabetes, n (%)	12 (36.4)
Hypertension, n (%)	29 (87.9)
Hyperlipidemia, n (%)	30 (90.9)
CAD, n (%)	21 (63.6)
Smoking history, n (%)	28 (84.8)
Previous revascularization, n (%)	13 (61.9)
CVA	3 (9.1)
Limb assessment	
Ankle-brachial index (treated Limb), mean (SD)	0.78 ± 0.15
Rutherford category, mean (SD)	2.5 ± 0.7
Lesion characteristics	
SFA, n (%)	30 (90.9)
Ostial	1 (3.3)
Proximal	4 (13.3)
Mid	23 (76.7)
Distal	2 (6.7)
Popliteal artery, n (%)	3 (9.1)
Proximal	1 (33.3)
Mid	1 (33.3)
Distal	1 (33.3)
Mean lesion length (cm)	5.5 ± 3.5
Reference vessel diameter, mm	4.7 ± 0.7
Minimum lumen diameter, mm	1.2 ± 0.7
Diameter stenosis, %	75.6 ± 13.6
Occlusion, n (%)	4 (12.1)
Stenosis, n (%)	29 (87.9)
Calcification, n (%)	
None	1 (3.0)
Mild	11 (33.3)
Moderate to severe	21 (63.6)

*CAD* coronary artery disease, *CVA* cerebrovascular accident, *SFA* superficial femoral artery

The mean lesion length treated was 5.5 cm and the average percent diameter stenosis of the lesions in this group of patients was 76% (moderate-severe calcification in over 60% of the 33 patients). Lesions in the superficial femoral artery constituted the majority (90%) of the treated segments. All lesions were successfully crossed with 2.0-mm Turbo-Elite laser catheter to create the pilot channel and subsequently treated with 8.0 ± 3.9 passes of the Turbo-Booster system which has the capability of going up to 60 mJ/mm at 80 Hz [7]. The index IVUS analysis was independently performed by the MedStar Research Institute Core Laboratory and there was a close correlation between the IVUS derived increase in lumen diameter and the diameter as measured by angiography.

### IVUS quantitative assessment findings

The post-procedure IVUS findings for this cohort of patients are outlined in Table 2. This analysis included grayscale pullbacks from two phases for 33 patients which met the inclusion criteria and were available for these quantitative dissection-scoring analyses. There was an average of 57 frames evaluated per pullback (minimum of 30 frames and maximum of 60 frames per pullback), giving a total of 3734 frames (1867 matched for pre-ablation (post channel creation) and post-ablation phases).

**Table 2:** Post procedure intravascular ultrasound findings (n = 27)

	Post-pilot channel, mean (SD)	Post-turbo-booster, mean (SD)	p value
Area (mm^2^)			
Lumen	4.3 ± 2.0	9.6 ± 3.2	< 0.0001
Plaque plus media	22.6 ± 6.9	20.4 ± 7.3	0.0042
EEM	27.0 ± 7.5	30.0 ± 7.5	< 0.0001
Volume (mm^3^)			
Lumen	65.2 ± 24.1	109.9 ± 33.9	< 0.0001
Plaque plus media	203.5 ± 61.7	192.9 ± 67.6	0.1619
EEM	268.7 ± 70.8	302.8 ± 74.5	< 0.0001

*EEM* external elastic membrane

The pilot-channel creation resulted mainly in mild intimal disruption in the majority of the cases (about 50%) and 1 in 5 frames following treatment with the Turbo-Booster system showed tears limited to the intima.

**Fig. 2:**
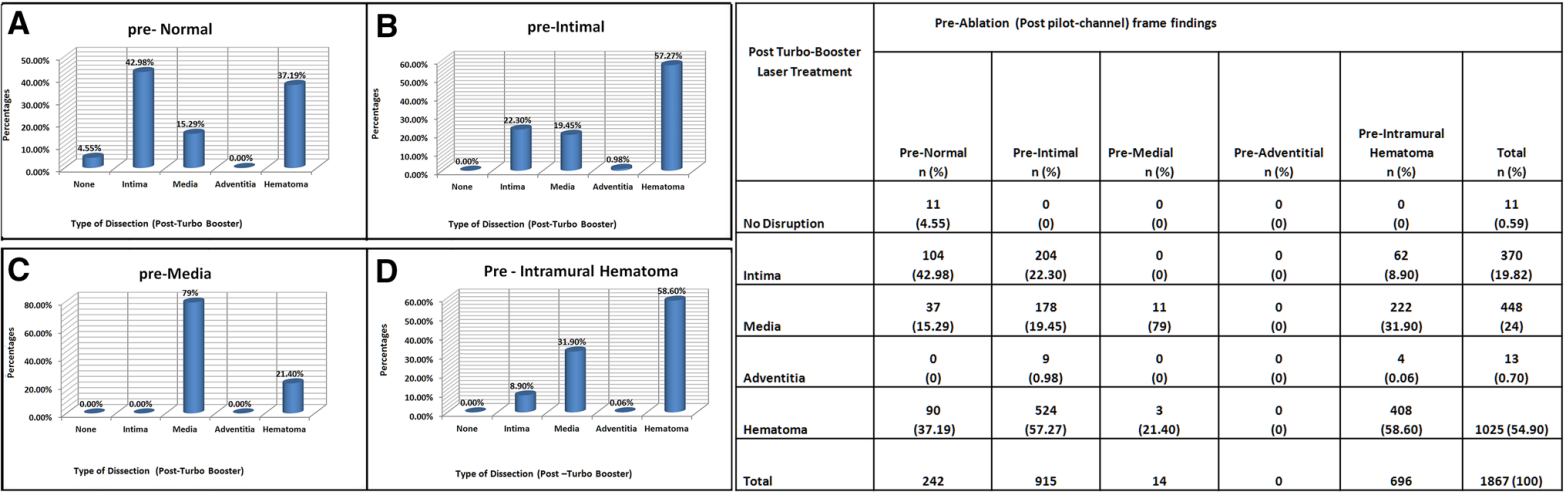
Post Turbo-Booster Laser Treatment Effect: 3 dimensional graphs illustrating the percentage of change from the pre-ablation (Post-Pilot channel creation with Turbo-Elite) state of the vessel wall to the degrees of dissection Post-Turbo Booster ablation. **a** Percentage of each class of dissection post-excimer laser treatment observed in IVUS frames with normal walls pre-ablation **b** percentage of each class of dissection post-excimer laser treatment observed in IVUS frames with some degree of intimal wall disruption pre-ablation. **c** Percentage of each class of dissection post-excimer laser treatment observed in IVUS frames with some degree of medial wall disruption pre-ablation. **d** Percentage of each class of dissection post-excimer laser treatment observed in IVUS frames with presence of intramural hematoma pre-ablation. Adjoining table showing frame level analysis of changes in the vessel wall pre- and post-Turbo Booster Laser treatment. No adventitial disruptions were present pre-ablation, hence there are no changes to report

IVUS quantitative analyses of vascular, luminal and plaque dimensions for this patient cohort pre- and post- laser treatment also showed that treatments with the Turbo-Booster system resulted in a significant increase in lumen area, 5.5 ± 3.2-mm^2^ (95% CI [4.3–6.8], p < 0.0001), reduction in plaque plus media volume (3 dimensions) of −10.6 ± 36.0 mm^3^ (95% CI [−25.8 to 4.6], p = 0.1619) and increase in the external elastic membrane (EEM) (Table 2) with mainly intramural hematoma formations observed in 55% of frames assessed and 24% medial dissections with less than 1% adventitial disruption (Fig. 2).

In the patient level analysis, 70% of patients’ pullbacks assessed showed evidence of disruptions extending up to the medial layer, among which only about 34% of the patients’ pullbacks showed evidence of medial injury in more than 20 frames (corresponding to a 10 mm arterial segment). We found adventitial disruptions in 9% of the 33 patients’ post-Turbo Booster runs, with none exceeding beyond a 4 mm segment involvement.

Marginal proportions of deep vessel (media and adventitial) disruptions between paired observations for pre-ablation and post-ablation phases were compared via the McNemar test. This showed a 0.75% adventitia-media post-Turbo-Elite compared to 24.69% Adventitia-Media post-Turbo-Booster (p value < 0.0001) Figure 2.

## Discussion

**Fig. 3:**
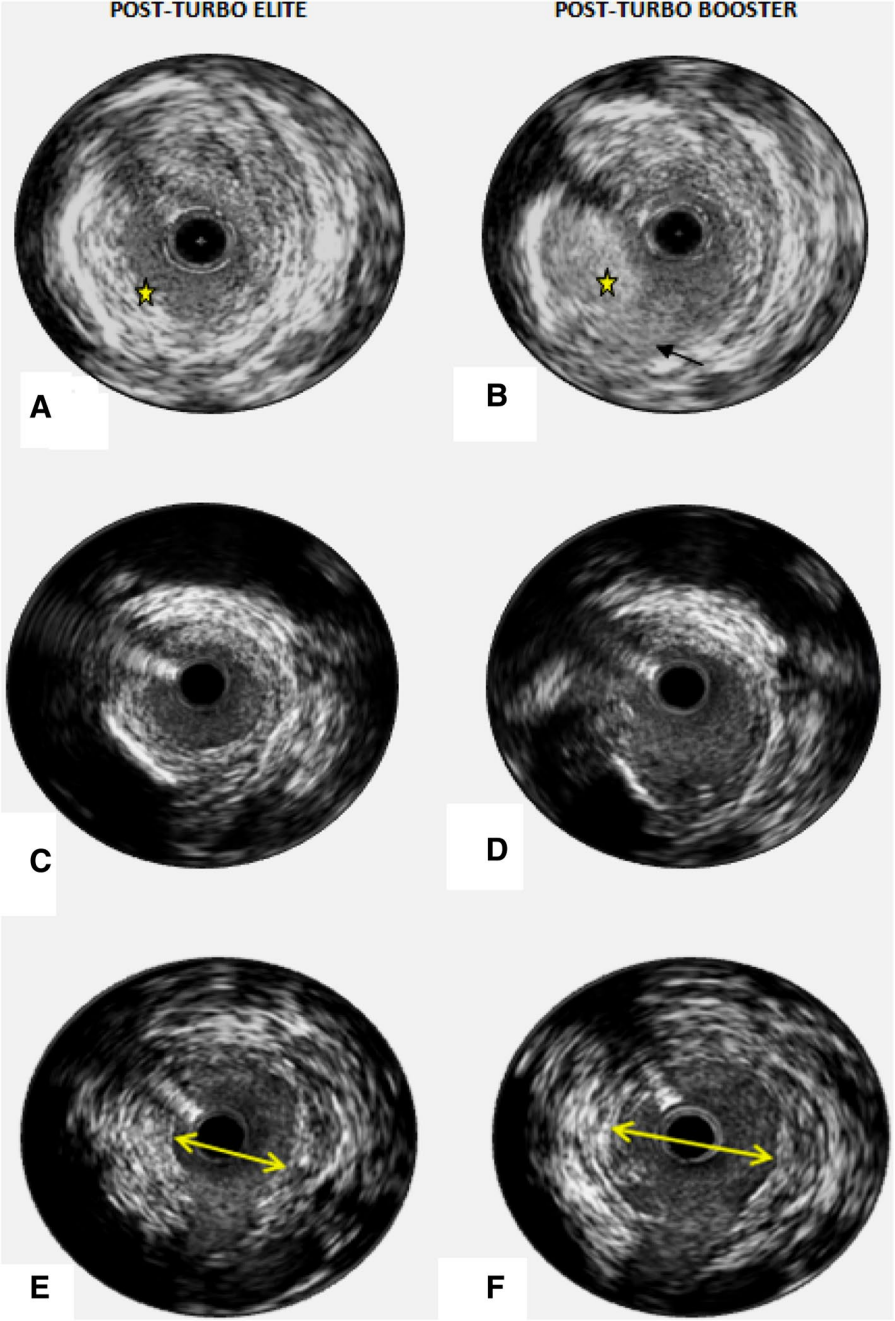
**a** Pre-ablation image following pilot channel creation with the 2-mm Turbo-Elite laser catheter; **b** post-ablation with the Turbo-Booster excimer laser system, with evidence of plaque debulking (star) between pre- and post with preservation on media (arrow); **c** and **d** Debulking of calcific plaque at 7′o clock between pre- and post-corresponding images. **e** and **f** Significant increase in lumen area between pre- and post- in a vessel area with concentric plaque

The major findings from this post hoc analysis of the CELLO registry patients are as follows (1) Treatment of femoropopliteal lesions with the Turbo-Booster laser system resulted in adventitial layer disruption in less than 1% of 1867 post ablation frames assessed by IVUS. (2) Intramural hematomas accounted for 50% of wall dissections observed and (3) effective debulking of plaque area was achieved with the excimer laser device. Our assessment showed that the 2 mm Turbo-Elite laser catheter mainly produced a minimal degree of disruptions while creating a channel through the lesion. Although we observed that most of these progressed to intramural hematomas of varying degree following Turbo-Booster debulking, these treatments produced significant reductions in calcific plaque (as identified by IVUS) (Fig. 3a–f).

### Excimer laser atherectomy and CELLO registry findings

The ability of the excimer laser to remove hyperplastic tissue by cutting through atheroma in the vessel lumen has been previously documented [8]. The excimer laser generates cool light in the ultraviolet (UV) spectrum. This energy is emitted from the tip of the catheter and breaks down hyperplasic tissue in atherosclerotic plaques within the diseased artery whilst the controlled ablation creates larger lumens with minimal thermal injury and less chance of distal embolization. Three distinct mechanisms of action contribute to laser photoablation: (1) photochemical disruption of cellular molecular bonds, (2) localized photothermal heat production that causes steam vapor disruption of cell membranes, and (3) photomechanical kinetic energy that dissipates cellular debris. Each laser pulse has duration of 125 nanoseconds and a depth of penetration of up to 100 μm [9].

The CELLO registry results showed remarkable evidence of significant tissue removal following laser ablation (reduction in diameter of stenosis from 77.3 to 42.5%) exceeding the targeted primary endpoint (defined by ≥ 20% average reduction in the percent (%) diameter stenosis, post-laser and prior to adjunctive therapy, based on angiographic core laboratory assessment and no major adverse cardiac events). Flow-limiting dissections were infrequent with a low rate of self-limiting dissections reported in the immediate post-procedure period. Target lesion revascularization was reported in 23.1% of the CELLO participants at one-year follow up.

While decreasing the volume of plaque via atherectomy has been shown to confer primary patency (with good procedural success in the short term), [10] there remains a growing concern over the loss of patency and increasing rates of revascularization at 12 months and beyond. Vessel wall injury/dissections, perforation thrombosis and distal embolization are some of the established complications of atherectomy in general [11]. The increase in the rates of restenosis at the long term has been linked to arterial wall injury during the initial procedure and the depth of injury is possibly a strong predictor of restenosis [4].

The exact mechanism of restenosis is not known, but evidence shows that it is mainly due to excessive neointima formation [12]. Any form of percutaneous intervention leads to some degree of mechanical injury that induces vascular inflammation, smooth muscle proliferation and extracellular matrix deposition with resulting neointimal thickening and restenoses [13]. Restenosis has previously been reported in up to 60% of primarily successful percutaneous interventions [14] but the rates of restenosis have varied across studies involving other atherectomy devices.

Zeller et al. [15] reported a 38.2% rate of restenosis based on duplex imaging among 172 patients-210 lesions (femoro-popliteal and infra-popliteal vessels) treated with Pathway Jetstream PV Atherectomy System while Keeling et al [16] reported a restenosis rate of 16.7% at 3 months(1-year primary and secondary patency of 61.7 and 76.4%, respectively), from their database of 60 patients in whom 70 plaque excisions were performed. More recently the COMPLIANCE 360 Trial noted freedom from TLR rates (plus adjunctive stenting) or restenosis of 77.1% (6 months) and 81.2% (12 months) post orbital atherectomy and balloon angioplasty [17]. Conversely, The DEFINITIVE AR Trial (Atherectomy Followed by a Drug Coated Balloon to Treat Peripheral Arterial Disease; ClinicalTrials.gov Identifier: NCT01366482) which was designed to evaluate the effect of treating a lesion with directional atherectomy (SilverHawk/TurboHawk, Medtronic Endovascular) followed by DCB (Cotavance, Medrad) versus DCB alone in 121 patients at ten centers reported a one-year target lesion percent restenosis was 33.6% in the atherectomy plus DCB group versus 36.4% in the DCB alone group.

Our IVUS based analysis revealed media and adventitial wall injuries in only 25% (less than 1% adventitial) of the 1867 IVUS frames evaluated. While primary patency rates (% DS, 50%) were 59 and 54% at 6 and 12 months, respectively in the original CELLO report, secondary patency was 100% at both 6 and 12 months.

### Intravascular ultrasound assessment

IVUS imaging has a resolution which enables evaluation of lesion and vessel morphology via determination of plaque and media areas in addition to measurements of the external elastic lamina and luminal diameters in the coronaries and peripheral vessels. IVUS has previously been used in measuring lumen gain following laser atherectomy [18]. Tarricone et al. showed that were no significant differences between IVUS and histopathology confirmed adventitial injury following atherectomy [19].

The histopathologic evidence-based vessel wall assessment of a 108 patient (with TransAtlantic Inter-Society Consensus (TASC) A/B femoropopliteal lesions cohort by Tarricone et al. following directional atherectomy showed patency rates of 53%, and an overall stenosis rates of 57% at 1 year, most of this accounted for by deeper wall injuries (97% with adventitia and media layer injuries and 11% with no injuries). Adventitial injury was shown in 55 patients (histopathology) and in 61 patients by IVUS.

Similar IVUS assessments have been done on a patient level in smaller number of patients/images [20]. Our frame-level based analysis was necessitated by the need to critically assess the arterial wall and accurately characterize the impact of the Excimer laser device on the vessel. The low rate of deeper wall injuries as shown in this post hoc IVUS analysis may translate to positive longer-term prognosis for patients treated with the Turbo device.

### Limitations

The percentage (50%) of complete IVUS images from the CELLO registry available for the assessment was relatively small. In addition, it would have been interesting to have imaging follow-up assessment in this patients to assess the fate of the changes observed in this post-hoc study.

## Conclusion

The Excimer laser based Turbo-Booster treatment of femoropopliteal lesions resulted in significant plaque debulking and increased lumen diameter with negligible degree of adventitial wall injury. As reported in the CELLO study, this treatment regimen achieved high procedural success and a > 1 grade decrease in the Rutherford category at each follow-up interval. Additionally, walking impairment scores and ABI were both improved at 12 months. When the results of this study are combined with the CELLO results, they suggest that treatments with the Turbo-Booster system are promising and help to prevent limb amputation over the 1-year follow-up.

## Abbreviations

CELLO: CliRpath Excimer Laser System to Enlarge Lumen Openings
SFA: Superficial femoral artery
IVUS: Intravascular ultrasound
ACC: American College of Cardiology
PAD: Peripheral arterial disease
FDA: Food and drug administration
FP: Femoropopliteal
MLA: Minimum lumen area
EEM: External elastic membrane
TASC: TransAtlantic inter-society consensus
CAD: Coronary artery disease
CVA: Cerebrovascular accident
